# Association of insulin-like growth factor-I with the severity and outcomes of acute ischemic stroke

**Published:** 2016-10-07

**Authors:** Masoud Mehrpour, Hessam Rahatlou, Negar Hamzehpur, Sahand Kia, Mahdi Safdarian

**Affiliations:** 1 Department of Neurology, School of Medicine, Iran University of Medical Sciences, Tehran, Iran; 2 Department of Neurosurgery, School of Medicine, Iran University of Medical Sciences, Tehran, Iran; 3 Department of Obstetrics and Gynecology, School of Medicine, Shahid Beheshti University of Medical Sciences, Tehran, Iran; 4 Department of Otolaryngology-Head and Neck surgery, School of Medicine, Tehran University of Medical Sciences, Tehran, Iran

**Keywords:** Acute Ischemic Stroke, Insulin-Like Growth Factor-I, Outcome, Severity

## Abstract

**Background:** The aim of this study was to evaluate whether higher serum levels of insulin-like growth factor-I (IGF-I) in the acute phase of ischemic stroke are associated with less severe strokes and better functional outcome in a period of 12-month follow-up.

**Methods:** From October 2014 to August 2015, patients with the diagnosis of acute ischemic stroke admitted to the stroke unit of Firoozgar Hospital, Tehran, Iran, entered this prospective study. National Institutes of Health Stroke Scale (NIHSS) and Modified Rankin Scale (MRS) for stroke scores were used to measure the severity and outcomes of an acute ischemic stroke at the time of admission and 1 year after the stroke, respectively.

**Results:** A total of 60 acute ischemic stroke patients (28 male, 32 female) with the mean age of 71.1 ± 9.0 years were evaluated for the serum level of IGF-I at the time of admission to the stroke unit of Firoozgar Hospital. There was seen a significant correlation between the IGF-I serum level and the MRS scores (P = 0.020; correlation coefficient = −0.32). IGF-I serum level had no significant correlation with NIHSS scores.

**Conclusion:** These results support that the higher serum levels of IGF-I at the time of stroke is associated with a significant better outcome in a 1-year period of follow-up. However, this hormone serum level seems not to have a predictable value for the ischemic stroke severity. Further studies are required to clarify the neuroprotective mechanisms of IGF-I in ischemic stroke process.

## Introduction

Cerebrovascular accidents had a great outbreak in human society that can lead to severe disability and reduced quality of life.^1^ Ischemic stroke is defined as a loss of brain function due to deficiency in the blood supply caused by arterial embolism or thrombosis.^2^ In Western countries, stroke is the most common cause of death after heart diseases and before cancers.^3^ Although reperfusion by tissue plasminogen activator (tPA) is the approved acute treatment of ischemic stroke, a very small proportion of patients benefits it due to strict inclusion criteria and the limited time for treatment.^4^

Insulin growth factor-I (IGF-I), a hormone with high molecular similarity to insulin, is known to be important in childhood growth and has anabolic effects in adults.^5^^,^^6^ Animal model studies reported IGF-I to play an essential role in the process of brain development.^7^ IGF-I is also capable to influence neuronal growth, excitability and release of the neurotransmitters.^8^ It is an endogenous factor for neurons survival, glial and endothelial cells and may enhance functional recovery after injury by stimulating the precursors of neural and oligodendrocyte to proliferate.^9^

Protective effect of this hormone in preventing nerve damages has been demonstrated in cultured neuronal cells,^10^^,^^11^ and the positive effect of exogenous administration of IGF-I in neurogenesis have been shown in mouse brain.^12^^,^^13^ This hormone is also effective in the survival of both motor and sensory neural cells.^14^ It has an effect in regulating neural development including neurogenesis, myelination, synaptogenesis, and dendritic branching after neuronal damage.^15^ IGF-I has been reported as a potent neuroprotective compound ischemic stroke studies of rodent models.^16^ A small study in elderly patients with stroke found an inverse relation between circulating IGF-I levels, determined within 24 hours of admission, and the outcome, mainly death.^17^ A relationship between improvement in functional and cognitive scores in revalidating stroke patients and higher IGF-I serum levels is also reported.^18^ IGF-I serum level has been reported to increase after treatment with tPA to 70% in stroke patients.^19^ Specific transport across the blood brain barrier has been reported for IGF-I^20^ and due its disruption after cerebral ischemia^21^ may support the hypothesis that systemic IGF-I could be considered as a new treatment goal in acute phase of stroke. 

A review by Kooijman et al.^16^ showed IGF-I to be neuroprotective in animal models of cerebral ischemia. Epidemiologic studies on human populations reported a higher rate of mortality in acute ischemic stroke patients in association with lower IGF-I serum levels^6^^,^^17^^,^^22^^-^^24^ and lower serum levels of IGF-I in patients with ischemic and hemorrhagic stroke is in relationship with 1.5 and 5.2 times more mortality rate, respectively.^25^ Some studies have shown that exogenous IGF-I reduces neurological damage and neural defects in the acute phase after cerebral ischemic stroke within 24 hours to 7 days.^26^^,^^27^

This cross-sectional study was designed to evaluate the effect of IGF-I serum level at the acute phase of stroke in the severity and outcome of the patients in a 12-month follow-up.

## Materials and Methods

The Local Ethics Committee of Firoozgar Clinical Research Development Center has approved this study. Informed consent was taken from the patients before collecting blood samples. The information of patients remained confidential and used only for research purposes.

Acute ischemic stroke patients admitted to the stroke unit of Firoozgar Hospital, Tehran, Iran, from October 2014 to August 2015 entered this prospective study. Stroke patients admitted with any of the following criteria were excluded from the study: age < 50, hemorrhagic stroke, previous brain trauma, insulin dependent diabetes, liver or kidney failures, Infectious disease, receiving any treatment that would affect growth hormone-IGF-I axis. The blood sample of the included patients was collected to evaluate the serum IGF-I levels (ng/ml) at the acute phase of stroke. IGF-I serum level was assayed with the method of chemiluminescence immunoassay after acid-ethanol extraction in the laboratory of Firoozgar Hospital during the first 24 hours after the ischemic stroke. 

To measure the severity and outcomes of acute ischemic stroke at the time of admission and 1 year after the stroke, the National Institutes of Health Stroke Scale (NIHSS) and Modified Rankin Scale (MRS) scores were used by an expert neurologist, respectively. NIHSS objectively quantify the severity and impairment caused by stroke based on clinical symptoms and disorders.^5^^,^^28^ While MRS determines the degree of dependence or disability caused by stroke.^29^^,^^30^

The collected data were analyzed by SPSS software (SPSS Icn., Chicago, IL, USA). Only those patients were included in the final analysis that completed the 1-year follow-up. To investigate IGF-I serum levels changes in all patients and for each group repeated measurement ANOVA test and post-hoc were used. Independent t-test, one-way ANOVA and chi-square were also used to compare qualitative and quantitative variables between groups. P < 0.005 was considered statistically significant.

## Results

A total of 60 patients with the mean age of 1.71 ± 9.0 years completed the 1-year follow-up and entered this cross-sectional study. 28 patients were male (46.7%), and 32 patients were female (53.3%).

**Table 1:** Categorization of the patients according to their IGF-I serum levels and MRS scores

**MRS**		**Number of patients (%)**	**Mean ± SD (ng/ml)**
No symptoms	10 (16.6)		234 ± 218	
No significant disability: Able to carry out all usual activities, despite some symptoms	6 (10.0)		140 ± 18	
Slight disability: Able to look after own affairs without assistance, but unable to carry out all previous activities	3 (5.0)		204 ± 230	
Moderate disability: Requires some help, but able to walk unassisted	5 (8.3)		130 ± 74	
Moderately severe disability: Unable to attend to own bodily needs without assistance, and unable to walk unassisted	13 (21.6)		128 ± 106	
Severe disability: Requires constant nursing care and attention, bedridden, incontinent	5 (8.3)		45 ± 27	
Dead	18 (30.0)		117 ± 115	

MRS: Modified Rankin Scale; IGF-I: Insulin-like growth factor-I; SD: Standard deviation

At the time of admission, the mean of IGF-I hormone serum levels was 110.0 ± 119.5 (ng/ml). Table 1 shows the categorization of the patients according to their IGF-I serum levels and MRS scores. There was seen a significant correlation between the IGF-I serum level and the MRS scores (P = 0.025; correlation coefficient = −0.329). While no significant correlation was found between the IGF-I serum level and NIHSS score (P = 0.346; correlation coefficient = 0.058).

## Discussion

This study shows that in 12-month follow-up, higher serum levels of IGF-I in the acute phase of ischemic stroke was associated with a better outcome in a group of 60 patients. However, it no association was found with the stroke severity. In the study by de Smedt et al., the association between IGF-I serum levels in 255 patients with ischemic stroke was evaluated with the outcomes and severity of stroke. In this study, we used NIHSS and MRS to define the progression and the severity of the stroke at the time of admission and 3 months later. After controlling the confounding factors, it has been shown that patients with higher IGF-I and IGF binding protein-3 serum levels within 6 hours of stroke had better functional and neurological outcomes 3 months later. In this study just like ours, stroke severity was not significantly associated with IGF-I serum levels and the authors concluded that better improvement after cerebral ischemic stroke is in association with the higher IGF-I serum levels.^9^ Bondanelli et al.^18^ studied 42 patients during rehabilitation after ischemic stroke to evaluate the relationship between serum levels of IGF-I with stroke severity and outcome. NIHSS was used to define the severity of stroke in this study and MRS, functional independence measure (FIM), and Los Amigos Cognitive Functioning Scale (LCFS) were used to determine the stroke progression. Similar to our findings, no significant association was reported between the ischemic brain damage severity caused by stroke and the IGF-I serum levels. While, using LCFS, FIM and MRS scores showed statistically better outcomes in patients with IGF-I serum levels more than 161.8 (µg/dl). This study also examined the adrenocorticotropic hormone, luteinizing hormone, follicle-stimulating hormone, and prolactin levels that showed no significant relationship with FIM and LCFS scales. Authors suggested that higher serum levels of IGF-I are associated with better recovery and better cognitive performance after cerebral ischemic stroke, which emphasizes the protective effect of this hormone for the nervous system. Our findings are also in consistency with the study by Denti et al.^17^ in which low levels of IGF-I were introduced in relation with worse neurologic function in 88 elderly patients 3 months after the stroke. In comparison to the control group, the mean of IGF-I serum levels was lower in ischemic stroke patients in this study. In addition, lower IGF-I levels were in correlation with poor outcomes. This study suggests IGF-I as a predictor of the stroke outcome in elderly patients. In a very important case–control study with more than 57000 patients and a 5-year period of follow-up, Johnsen et al.^6^ reported a 2-fold increased risk of ischemic stroke in patients in bottom quartile of IGF-I serum levels compared with those in the upper quartile. This valuable finding can support the hypothesis of the IGF axis involvement in the pathogenesis of ischemic stroke.

Some limitations of this study should be taking into consideration. First, generalizability of the results of our study is limited by small sample size; however, the same findings are noticed in almost all similar studies. On the other hand, MRS may be assumed as an insensitive measure of functional outcome and more accurate objective outcome measures may be used for further studies. On the other hand, it might be better to report the correlation between IGF-1 and 3-month and/or 6-month outcomes in addition to 1 year follow-up. 

## Conclusion

This study was however the first of its kind in Iranian population. From the findings of this study and comparison with other similar studies, we can consider that there may strongly be an association between neurological function outcome after the acute ischemic stroke and IGF-I serum levels. Although, this hormone seems not to be associated with the severity of cerebral ischemic stroke. This study recommends the probable effect of IGF-1 administration on stroke outcome. Further randomized control trials are required to assess the effect of IGF-1 administration on stroke outcome in addition to the neuroprotective mechanisms of this hormone.

## Notes:

**How to cite this article:** Mehrpour M, Rahatlou H, Hamzehpur N, Kia S, Safdarian M. Association of insulin-like growth factor-I with the severity and outcomes of acute ischemic stroke. Iran J Neurol 2016; 15(4): 214-8.
